# The Control of Mesenchymal Stromal Cell Osteogenic Differentiation through Modified Surfaces

**DOI:** 10.1155/2013/361637

**Published:** 2013-05-22

**Authors:** Niall Logan, Peter Brett

**Affiliations:** Biomaterials and Tissue Engineering, Eastman Dental Institute, University College London, 256 Gray's Inn Road, London WC1X 8LD, UK

## Abstract

Stem cells continue to receive widespread attention due to their potential to revolutionise treatments in the fields of both tissue engineering and regenerative medicine. Adult stem cells, specifically mesenchymal stromal cells (MSCs), play a vital role in the natural events surrounding bone healing and osseointegration through being stimulated to differentiate along their osteogenic lineage and in doing so, they form new cortical and trabecular bone tissue. Understanding how to control, manipulate, and enhance the intrinsic healing events modulated through osteogenic differentiation of MSCs by the use of modified surfaces and biomaterials could potentially advance the fields of both orthopaedics and dentistry. This could be by either using surface modification to generate greater implant stability and more rapid healing following implantation or the stimulation of MSCs *ex vivo* for reimplantation. This review aims to gather publications targeted at promoting, enhancing, and controlling the osteogenic differentiation of MSCs through biomaterials, nanotopographies, and modified surfaces for use in implant procedures.

## 1. Introduction 

Biomaterials have advanced significantly in recent years, although with an aging global population, what was once deemed to be an acceptable longevity for an implant is no longer so, driving the development of improved biomaterials that have superior performance and greater longevity. One area where this effect is very obvious is in orthopaedic arthroplasty. It is estimated that by 2030, there will be a 174% increase in the need for total hip replacements (THR) accompanied by a 674% rise in total knee replacements (TKR) [1]. Over the same time period, the need for revision surgery, to surgically replace or repair a failing prosthetic joint, is also set to increase dramatically. To help reduce the need for revision surgery, suitable biomaterials must be developed for these applications based on their mechanical and biocompatible properties [2]. Ideally for orthopaedic implants, the material must be mechanically strong enough to tolerate the load of the joint whilst also having a Young's modulus that is suitable to transfer load into the surrounding tissues. It is also important for the implant material to be bioinert to prevent any inflammatory response, although bioactive materials are currently the implant material of choice [3], as they can promote positive biological responses such as osseointegration. Osseointegration is important for bone healing and is the formation of a direct interface between an implant and bone without the need for connective soft tissue [4, 5]. For the formation of a direct interface to occur between an implantable biomaterial and native bone tissue, the recruitment of cells with osteogenic potential must happen on the surface of the implant. Colonisation by such cells to the site of interest occurs through the release of growth factors and cytokines into the clot surrounding the site of implant placement, and it is widely accepted that MSCs are the first osteogenic cells recruited to such sites *in vivo* [6, 7]. MSCs are multipotent cells that have the ability to self-replicate and to differentiate into osteogenic, chondrogenic, adipogenic, fibroblastic, and neural lineages [8]. Through the release of local factors and interaction with the biomaterial surface, MSCs ideally can be triggered to differentiate along their osteogenic lineage, forming osteoblasts capable of facilitating bone healing at the implant site. This is triggered by a complex combination of events, involving cytoskeletal tension within the cells, cellular membrane signalling, focal adhesions, and the secretion of a calcium rich extracellular matrix (ECM). To improve this cellular response, the implant surface can be tailored with topographical and chemical cues, with factors that have been shown to modulate the differentiation of MSCs, including both micro- and nanoscale surface topographies, along with surface energy and chemistry. This review aims to examine the use of modified surfaces to trigger and enhance the osteogenic differentiation of MSCs, with the ultimate aim of creating orthopaedic and dental implants that support osseointegration and promote more rapid healing. 

## 2. Titanium

Titanium has long been the gold standard for orthopaedic and dental implants due to its excellent biocompatibility, corrosion, and wear resistance and its ability to promote osseointegration at the bone-implant interface [9]. What helps to trigger the osteogenic differentiation of MSCs on titanium has been heavily investigated, resulting in the formation of titanium alloys with modified surfaces aimed at promoting osseointegration. A titanium implant material with a modified surface from Institut Straumann AG (Waldenburg, Switzerland), known as SLActive, has been extensively researched in recent years as a method of promoting the osteogenic differentiation of MSCs and in turn leading to greater levels of osseointegration being observed after implantation. The novelty of the surface is that it utilises two elements that have been shown to be important in engendering good bone responses; the first is a rough topography optimised for bone response [10], and the second is high surface energy rendering the surface superhydrophilic [11, 12]. Buser et al. first demonstrated the bone integration potential of SLActive in an animal study using a miniature pig model. Through bone implant contact (BIC) measurements, SLActive was shown to promote enhanced bone healing at early stages compared to the nonhydrophilic roughened surface (SLA) [13]. In similar studies, Schwarz et al. used a canine model to investigate various levels of bone integration and again found SLActive superior in the level of bone healing observed [14–16]. Bornstein et al. utilised a canine model to investigate bone apposition to both SLActive and SLA implants. The SLActive surface demonstrated significantly more bone apposition after two weeks of healing, highlighting the surfaces ability to promote accelerated early osseointegration [17]. In human studies, Lang et al. performed a dental study involving 49 patients. Implants were inserted into the retromolar area and following retrieval; the level of osseointegration observed was superior at 2 and 4 weeks for SLActive [18]. To gain further understanding of bone integration on implants at a genetic level, Donos et al. implanted both SLActive and SLA implants in the retromolar area of nine human patients. Upon retrieval, analysis of RNA extracted from the tissues attached to the implants was performed. SLActive displayed an upregulation in genes associated with both osteogenesis and angiogenesis 1 week after implantation. It was concluded that the proosteogenesis and proangiogenesis influence of the SLActive surface may be responsible for its early osseointegrative superiority [19]. For a more in-depth review regarding SLActive *in vivo* studies, please refer to Schwarz et al.'s recent publication [20]. 

These *in vivo* studies demonstrate the bone integration potential of the SLActive surface although failing to identify the underlying biological reasoning behind its superior osseointegration properties. As MSCs have been identified as a key cell type necessary for osseointegration and bone healing [6, 7], the cellular response of human MSCs to SLActive surfaces, in particular their osteogenic differentiation, was highlighted as an area of importance. This was initially studied by Wall et al. [21] who utilised both SLActive and SLA surfaces, along with a smooth titanium surface as a control (SMO). Wall et al. found that both SLActive and SLA outperformed SMO [21], which was expected taking into account the difference in surface roughness, previously highlighted as an important factor for bone integration [10]. Levels of RNA for the osteogenic genes WNTa, BSP, Runx2, and SPP1 were analysed, and SLActive was observed to express higher levels of RNA expression for the osteogenic markers examined. The higher levels of RNA found on the SLActive surface point to an accelerated osteogenic response from the MSCs and support the* in vivo* genetic study discussed earlier [19]. 

In an attempt to further understand the mechanisms involved between microstructured titanium surfaces such as SLActive and the osteogenic differentiation of human MSCs, Olivares-Navarrete et al. performed a study identifying and analysing key markers believed to be important in osteogenic differentiation [22]. It was found that multiple osteogenic markers were significantly increased on the SLActive surface, including alkaline phosphatase (ALP), a hydrolase enzyme responsible for supplying calcium nucleation sites with inorganic phosphate, and early marker of osteogenic differentiation [23]. Osteocalcin (OC), osteoprotegerin (OPG), and transforming growth factor beta 1 (TGF-*β*1), late markers for osteogenic differentiation, were also significantly increased on SLActive, although vascular endothelial growth factor A (VEGF-A) was observed to be significantly decreased. The expression of osteogenic genes was also analysed, and SLActive was found to upregulate Runx2, OC, ITGA2, and ITGB1 supporting previous publications [19, 21]. Olivares-Navarrete et al. went on to investigate coculturing combinations of MSCs with MG63 cells in an attempt to examine if contact with osteoblast-like cells would have a real-time effect on the MSC differentiation observed on the Ti surfaces. This experiment aimed to mimic the *in vivo *environment allowing for cross-talk to occur between some of the cell types that would be present in areas of bone healing. It was found that local factors released by MG63 cells only had an effect on the osteogenic phenotype of MSCs grown on the SLA and SLActive surfaces. When MSCs were cocultured with ITGA2 silenced cells, a reduction in the level of osteogenic differentiation was observed on all surfaces, supporting previous work which highlighted the importance of ITGA2 to osteoblast response on Ti substrates [24]. DKK2 silenced cells were also cocultured with MSCs producing similar results on the SLActive and SLA surfaces, with reduced levels of osteogenic differentiation being observed from the silenced cell culture. This also underlined the importance of this factor for the osteogenic differentiation of MSCs on Ti substrates [25]. 

To further examine the SLActive surface, Khan et al. performed a study investigating certain key bone matrix components of human MSCs thought to be important for osteogenic differentiation [26]. Khan et al. hypothesised that a measure of the level of osteogenic differentiation occurring could be related to the level of calcium and collagen deposition in the ECM. The important finding from this study showed that the amount of calcium deposited per ng against type 1 collagen was significantly higher on SLActive suggesting that the quality as well as quantity of bone deposited was superior on SLActive. The ECM has emerged as a strong factor in the control of stem cell fate through physical interactions [27–29], implying that the enhanced osseointegration properties of the SLActive surface [13–18] may be due to the stiffness of the calcified ECM [26] human MSCs produce on the SLActive surface. Khan et al. also studied ALP expression, the genetic expression of osteogenic genes Runx2, osteopontin (OP) and bone sialoprotein type 2 (BSP2), and osteogenic protein expression of OC, OPG, and growth differentiation factor 15 (GDF-15). ALP expression and early upregulation of osteogenic genes Runx2, OP, and BSP2 at day 1 again support the evidence that the SLActive surface promotes an enhanced osteogenic genetic response from MSCs [19, 21, 22, 30]. Osteogenic protein expression was analysed over a three-week time course, and both OPG and GDF-15 were observed to be significantly higher at 3 weeks on SLActive [26]. 

## 3. Nanotopography

 Micron scale topographies have been shown to influence the lineage-specific differentiation of MSCs [21, 22, 26], and further studies are looking to increase cellular response and control. The recent emergence of the use of nanotopographies to control and enhance the differentiation of MSCs holds great potential in the fields of both orthopaedics and dentistry. The ability to create various topographies in either ordered or random formats that can be easily modified to specific sizes opens up a vast number of possible uses. Topography-mediated fate determination has numerous advantages over other techniques used to drive lineage-specific differentiation. These include an increase in durability over surface chemistry modification and the removal for the need of synthetic or biological moieties to direct differentiation, which have significant regulatory issues for use *in vivo*. Surfaces such as the previously discussed SLActive are on the micron scale in terms of roughness and topography. Nanotopographies have a distinct advantage over such surfaces as they have the ability to replicate the features of tissues found *in vivo* on the nanoscale. Bone tissues have features that range from the macro- (osteoids), micro- (mineralised structures 0.8–1.4 *μ*m [31]), and nanoscales (collagen fibre bundles 5–10 nm with 67 nm striation and HA crystals 225 nm [32]). At the nanoscale of bone, a complex combination of protrusions, fibres, and topographical pits is present and through interaction with this topography *in vivo*, an osteogenic response can be triggered in MSCs, allowing for the formation of new bone tissue to occur. The research into materials nanotopography aims to replicate this *in vivo* MSC stimulation, controlling the lineage-specific differentiation of MSCs solely through cellular interactions with material surface features on the nanoscale.

## 4. Polymer and Silicon Nanotopographies 

Electron beam lithography (EBL) patterned substrates of polymethylmethacrylate (PMMA), with 120 nm diameter and 100 nm deep nanopits, were analysed for their effects on osteogenic MSC fates by Dalby et al. [33]. Ordered patterns included square (SQ) and hexagonal arrays (HEX), with disordered patterns including square arrays with pits placed randomly by up to 50 nm (DSQ50) and 20 nm (DSQ20) on both axes from their position in a true square array. A final disordered pattern had pits placed randomly over a 150 *μ*m by 150 *μ*m field (RAND). After 21 days in culture, it was noted that MSCs on the DSQ50 surface showed areas of early nodule formation and intense cell aggregation, while exhibiting regions positive for bone-specific ECM proteins, OP and OC. At 28 days, mineralisation occurring within nodules was positively identified, and significantly this only occurred on the DSQ50 surface. The highly ordered surfaces (SQ & HEX) showed decreased levels of osteoprogenitor cell density, and MSCs were fibroblastic in appearance. To further study the osteogenic potential of the DSQ50 surface, Dalby et al. performed RNA analysis on MSCs cultured on the DSQ50 surface, against a planar control and a planar material with the corticosteroid dexamethasone, a chemical additive used to stimulate the osteogenic differentiation of MSCs. Cells cultured with dexamethasone had the highest levels of upregulation with 24 gene hits, although the DSQ50 surface was able to trigger 11 gene hits in comparison to the controls 3 hits. Gene expression analysis was then performed for adhesion molecule ICAM1, signalling molecule receptor TGFBR1 and the osteogenic genes OCN and ALP. As before, the DSQ50 surface was able to significantly upregulate these important osteogenic genes, although not to the same level as the dexamethasone-supplemented cells. Dalby et al. were able to demonstrate for the first time that disordered nanotopographies (DSQ50) had the potential to enhance the osteogenic differentiation of MSCs in the absence of chemical stimuli.

In a follow-up study, Dalby et al. looked at the genomic expression of human MSCs on two different nanotopographies created on PMMA [34]. Firstly, surface 40 : 400 was created via photolithographically producing pits in PMMA, of dimensions 400 nm depth and 40 *μ*m diameter, comparable to that of osteoclastic resorption pits found *in vivo*. Secondly, via polymer demixing, a surface referred to as 3% : 3000 was created, producing polymer islands of 33 nm in height, a surface modification technique previously identified as being able to stimulate a response from various cell types [35]. It was found that the topographical surfaces were able to alter the genomic profile of the MSCs in a similar way to that of dexamethasone. It was noted that the 3% : 3000 surface outperformed the 40 : 400. Interestingly some topography only pathways were noted, such as p38 mitogen-activated protein kinase (MAPK), actin cytoskeletal signalling, fibroblast growth factor (FGF), and platelet-derived growth factor (PDGF) [34]. Upregulation of genes ICAM1, TGFBR1, OCN, and ALP was also observed in MSCs on the test topographies, supporting Dalby et al.'s earlier observations from the DSQ50 surface, which also initiated an upregulation in some of these key genes [33].

In a similar study by Biggs et al., PMMA embossing was used to create disordered nanotopographies of nanocraters and nanoislands, alongside ordered nanotopographies of pits arranged in square and hexagonal arrays [36]. Nanocraters were approximately 47 nm in depth with 3 *μ*m diameter, while the nanoislands were approximately 45 nm in height and 3 *μ*m diameter. The ordered arrays were shown to possess pits of 120 nm diameter with a depth of 100 nm, possessing a centre-centre spacing of 300 nm. Human MSCs that had been STRO-1 positively enriched were analysed after being cultured on the substrates, and it was shown that changes in the genetic expression of the cells were occurring due to the topographical features of the substrates. Cell integrin signalling and morphology were affected by all the substrates, although microarray data regarding the greatest number of modulated gene functions for canonical signalling pathways was observed from the nanopit arrays. Transforming growth factor beta (TGF-*β*) was shown to be upregulated on all substrates, which for nanoisland substrates was coupled with a minor upregulation in TGF-*β*-activated kinase-1 binding protein (TAB1) and Ca^2+^/cAMP-response elements binding protein (CPB).

In a follow-up study, Biggs et al. investigated the effect nanotopographies have on the ERK/MAPK signalling pathway for STRO-1+ human MSCs [37]. The signalling cascade, extracellular signal-regulated kinase (ERK), is a known member of the MAPK pathway, shown to play a significant role in MSC differentiation [38–40]. Intricate signalling pathways, such as the ERK/MAPK pathway, play a vital role in the cellular differentiation of MSCs through translating tensions applied at the tissue level to cellular functions. Biggs et al. investigated four PMMA nanotopographies; two nanopit arrays (SQ & HEX) as previously described [36], and two groove/ridged arrays, approximately 300 nm deep with 10 *μ*m and 100 *μ*m widths, respectively. It was found that the ordered nanopit arrays caused a downregulation in the expression of multiple signalling molecules, supporting previous research that highly ordered PMMA arrays restrict the osteogenic differentiation of MSCs [33]. MSCs cultured on the 10 *μ*m grooves displayed both up- and down-regulations of signalling molecules, although upregulation in the expression of focal adhesion kinase (FAK) as well as MAPK phosphatases (MKP) was noted. The 100 *μ*m grooved substrate caused widespread upregulation in the genes associated with the ERK/MAPK pathway, alongside significant production of osteocalcin. Biggs et al. were able to demonstrate through the use of grooved, ridged nanosubstrates that the ERK/MAPK signalling pathway could be stimulated for the osteogenic differentiation of human MSCs [37].

The modulation of human MSC osteogenic differentiation through topographically patterned ridges and grooves on substrates was further investigated by Watari et al. [41]. Utilising silicon substrates, three topographies were studied, each with grooves 300 nm in depth, but with 400 nm, 1400 nm, and 4000 nm pitch, respectively. The genetic expression of Runx2, a master switch for osteogenic differentiation of MSCs [42], was studied after three days in culture, and it was found that the 400 nm pitch surface stimulated significantly higher levels of expression than that found on the 4000 nm surface and planar control. A marker for mature osteoblasts, OCN, was also examined, and there was a significant topographical effect in comparison to the planar control. Over a three-week time course, it was shown that the 400 nm pitch surface had significantly higher levels of calcium deposition at days seven and fourteen against the control, a trend shown before on the larger micron scale with the SLActive titanium surface [26]. To further examine the effect of the topographical surfaces, MSCs were then cultured on the substrates using traditional osteogenic media. The 400 nm substrate again displayed superior osteogenic properties, with significantly higher levels of calcium deposition at day 7 and higher Runx2 expression at day 3, in comparison to the control. Levels of OCN were again observed to be higher for topographical substrates in comparison to the control. In osteogenic media, it was shown that the 400 nm substrate was able to significantly increase the level of expression of ID1, a target gene induced by bone morphogenetic proteins (BMPs) [43] belonging to the TFG-*β* superfamily thought to play an important role in osteogenic differentiation of MSCs [44]. This trend was also evident in nonosteogenic media although not deemed statistically significant. Watari et al. were able to demonstrate the osteogenic potential of the 400 nm pitch surface [41], and it was suggested that the surface may have been superior due to its sizable comparison to the parallel array of type 1 collagen fibrils located in bone tissue *in vivo *[45]. It is important to note that Watari et al.'s conclusions differed from a previous publication by Yim et al., where grooved and ridged polymer substrates were shown to cause neural specific differentiation of human MSCs [46], although different coating materials and culture media were employed.

In an attempt to investigate the effect of micro- and nanoscale pillars on MSC differentiation, Brammer et al. created topographies on gold-coated silicon substrates [47]. Unlike the previous studies, the substrates were rendered hydrophobic instead of the primarily polymeric and hydrophilic surfaces. Photolithography and wet etching techniques were used to create micropillar (2.5 *μ*m height, 2 *μ*m diameter) and nanopillar (2.5 *μ*m height, 20 nm diameter) topographies, both with spacing proportional to their pillar diameters. Subsequently, gold deposition was then applied to keep the surface chemistry constant and focus on the topographically induced cell response. The nanopillar topography was shown to significantly affect the growth of the rat MSCs while also influencing stem cell fate. MSCs on the nanopillar topography were shown to have significantly greater adhesion at 2 hours and increased proliferation at 24 and 48 hours. MSC spreading was hindered at initial stages, although at three weeks in culture, dense cell aggregates had formed on the nanopillar surface, and it was hypothesised that the cells were differentiating due to the morphological changes. This hypothesis was based on work by Tang et al. who studied the relationship between cell-cell interactions and MSC osteogenic differentiation, where it was found that a positive linear relationship existed between the two [48]. The differentiation potential of Brammer's nanotopographies was studied, including markers for adipogenesis, chondrogenesis, and osteogenesis. It was found that the nanopillar topography had a higher osteogenic potential through significantly enhancing the levels of osteogenic mineralisation, detected by alizarin red staining without the presence of chemical-inducing additives. High levels of the osteogenic-specific protein OP detected in the cell aggregates confirmed the dominant osteogenic properties of the nanopillar topography. 

## 5. Metallic Nanotopographies

As previously discussed, it has been shown that through the use of nanotopographical influence alone, the osteogenic differentiation of MSCs can be triggered and stimulated [33, 34, 36, 37, 41, 47], although the application of such surfaces still requires further research, in particular, the transmission of these influential topographies onto load-bearing implant materials with mechanical and biocompatible properties suitable for dental and orthopaedic application. As titanium is regarded as the gold standard for orthopaedic and dental applications [9] due to its excellent biocompatibility and mechanical properties similar to those of bone, many research groups have targeted the combination of titanium with nanotopographies tailored for lineage-specific differentiation of human MSCs.

Sjöström et al. studied the interaction of human MSCs on titania nanostructures produced on titanium surfaces via anodisation through a porous alumina mask [49]. Different topographies were created via increasing the anodisation voltage, resulting in three different surfaces exhibiting nanopillar structures of 15 nm, 55 nm, and 100 nm in height, respectively, (diameters 28 nm, 41 nm, and 55 nm). The topographies created at lower voltages, that is, the 15 nm and 55 nm surfaces, were described as having densely packed dot-like structures, in comparison to the 100 nm surface, which had pillar-like structures with greater centre-centre distances. The MSC response on the surfaces showed that the 15 nm high structures sustained well-spread cells with highly organised cytoskeletons and many large focal adhesions thought to be important for osteogenic differentiation of MSCs [50, 51]. In comparison to the other topographies, the MSC cytoskeletons were less organised with fewer and smaller focal adhesions. Immunohistochemical staining for osteogenic markers OC and OP was performed after 21 days in culture, and it was found that the 15 nm high structures exhibited bone matrix nodules rich in both OP and OC. The amount of bone matrix nodules observed decreased as the titania structures increased from 15 nm to 100 nm, respectively. 

To further examine the osteogenic ability of the 15 nm structured titanium surface, McNamara et al. performed a study similar to Sjöström et al. [49], although included additional techniques aimed at progressing the published literature [52]. The 15 nm structured titanium surface was studied against two other structured surfaces, with 55 nm and 90 nm heights, respectively, alongside a planar control. Again it was shown that the 15 nm structures promoted superior focal adhesions and an enhanced production of osteocalcin after 21 days in culture. The transcriptional factor Runx2, known to be important for the osteogenic differentiation of MSCs [42], is regulated by differential phosphorylation. After 2 days in culture, it was found that the level of phospho-Runx2 (pS456) in the nuclei of MSCs on the 15 nm surfaces was enhanced, resulting in an increase in the expression of this important osteogenic factor. Metabolomics, which studies the metabolic response of the MSCs, found that a substantial number of metabolic pathways were upregulated in MSCs on the 15 nm surface. These included pathways for amino acids, linoleic acid, and the functional pathway for ALP, known to play an important role in bone mineralisation [23].

Lavenus et al. studied the influence of nanopores on the differentiation of human MSCs and the level of osseointegration observed in an *in vivo* rat model [53]. Titanium surfaces were created with nanopores of 20 nm, 30 nm, and 50 nms via anodisation at 5 V, 10 V, and 20 V in a combination of acetic and fluorhydric acid. To identify the osteogenic ability of the nanotopographies, osteoblastic gene expression was studied without the presence of osteogenic supplements, and it was found that the Ti30 and Ti50 surfaces promoted early osteogenic differentiation of the MSCs through up regulation of Runx2 and Col1A1. The nanosurfaces also seemed to modulate osteogenic factors ALP, OCN, and BSP. In the *in vivo* section of Lavenus et al.'s research, osseointegration of nanostructured implants was studied in a rat tibia model. After 1 week, it was found that bone healing was significantly enhanced on nanostructured implants. Bone tissue was observed to be in direct contact with the implant; in comparison to the Ti control, a gap of 100 *μ*m was present between the implant surface and host bone tissue. At week 3, bone contact was clearly evident on the nanostructured implants with thick trabeculae following the contours of the surfaces, with the Ti50 surface appearing to have the highest quantity of bone. BIC values and pull out tensile tests confirmed the superiority of the Ti30 and Ti50 surfaces, implying that faster bone healing may have occurred around these nanostructured implants. Lavenus et al.'s work is novel for the fact it combines both *in vitro *and *in vivo *results on nanotopographies shown to be influential on both the osteogenic differentiation of MSCs and *in vivo *osseointegration. Although it would have been ideal to study both *in vitro *and *in vivo *tests from the same subject, this work still shows the osteogenic ability of nanopored titanium surfaces and may be extremely influential in future dental and orthopaedic implants. 

Zhang et al. looked at the behaviour and regulatory effects of rat MSCs on titanium substrates biofunctionalised with nano-sawtooth structures [54]. Hydrogen peroxide (H_2_O_2_) was used to fabricate two surfaces with nano-sawtooth structures different in dimensions, referred to as Ti-6 and Ti-24. Treatment of H_2_O_2_ for 6 hours produced nano-sawtooth structures with approximate widths of 10 nms and gap distances (distance between nano-sawteeth) of 100–200 nm. An increased treatment time of 24 hours produced the second surface, which displayed nano-sawtooth widths of 30 nm and gap distances of 200–300 nm. Under high magnification, both surfaces showed an interconnected, entangled network of nanostructures. Cell adhesion and proliferation were looked at first, and it was found that the large nano-sawtooth Ti-24 surface had significantly more adhesive cells present than on the Ti-6 surface and smooth Ti control. Proliferation was also observed to be enhanced on the Ti-24 surface at time points 4 and 7 days. The osteogenic ability of the nanosurfaces was analysed by observing mineralisation, together with osteogenic gene and protein expression. ALP was shown to be more positively pronounced on the nanosurfaces, alongside enhanced calcium deposition, with the most mineralised matrix being observed on the large nano-sawtooth Ti-24 surface. Osteogenic genes Runx2, OCN, and OPN were analysed using real-time qPCR, and it was shown that there was an upregulation from days 5 to 10 on the nanosurfaces in comparison to the Ti control, with the large nano-sawtooth Ti-24 surface promoting the greatest upregulation. Supporting the genetic findings, the expression of osteogenic proteins OP and OC was shown to be upregulated on the nanosurfaces, being strongest on the Ti-24 surface. Using rat MSCs, Zhang et al. were able to demonstrate that nano-sawtooth structures on titanium, in particular the Ti-24 surface, have the ability to regulate osteogenesis through enhancing adhesion, proliferation, and multiple osteogenic factors involved in differentiation.

The use of TiO_2_ nanotubes to control the lineage-specific differentiation of MSCs was studied by Oh et al., where it was found that different nanotube dimensions promoted drastic changes in human MSC behaviour [55]. TiO_2_ nanotubes were fabricated on Ti substrates via anodisation, creating surfaces with 30, 50, 70, and 100 nm nanotube diameters, respectively. It was found that smaller diameter nanotubes elicited cell adhesion but lacked differentiation ability. In comparison, larger nanotube surfaces promoted cell elongation and osteogenic differentiation, confirmed by positive staining for OPN and OCN after 21 days in culture, alongside quantitative real-time PCR gene expression of osteogenic markers OCN, OPN, and ALP after 14 days incubation. Oh et al. concluded that the larger diameter TiO_2_ nanotube surfaces (70 nm and 100 nm) were superior for osteogenic differentiation of MSCs by inducing cytoskeletal stresses through cellular elongation, which were then interpreted into internal biological responses, triggering osteogenesis within the cells. As the use of nanotopographies to control and enhance MSC differentiation is an emerging field of research, it is unsurprising that different responses to nanosurfaces have been published. In particular for TiO_2_ nanotube modified surfaces, Oh et al. reported an optimum diameter of 100 nm [55], where Park et al. have observed cell apoptosis at this size [56] and identified TiO_2_ nanotubes with a diameter of 15 nm to be optimal for cell adhesion and differentiation [57]. In an attempt to further improve the response of MSCs on TiO_2_ surfaces, functionalisation of the surface nanostructures with growth factors is currently being studied. Park et al. found that bone-morphogenetic-protein-2- (BMP-2-) coated TiO_2_ nanotube surfaces of 15 nm and 100 nm, respectively, caused a significantly different cell response [58]. BMP-2-coated 100 nm TiO_2_ nanotubes promoted chondrogenic differentiation of MSCs, in comparison to the 15 nm BMP-2-coated nanotubes which enhanced osteogenic differentiation. Lai et al. performed a similar study with TiO_2_ surfaces (30, 60 and 100 nm) functionalised with BMP-2, although all surfaces were able to enhance the osteogenic ability of the surface in comparison with identical uncoated surfaces [59]. Modification of titanium with TiO_2_ nanotubes holds great potential for influencing MSC behaviour and response, although further research is required considering the amount conflicting publications. For an in-depth review regarding TiO_2_ nanotubes for bone regeneration, please see Brammer et al.'s recent publication [60]. 

As nanotubes have been identified as an area of interest in nanotopographies, their combination with microtopographies has recently been investigated. Zhao et al. used rat MSCs to examine the level of osteogenic differentiation occurring on titanium substrates fabricated with micropitted/nanotubular titania topographies [61]. In an effort to imitate the arrangement of natural bone ECM, several topographies were created via acid etching and/or anodisation. Anodisation of titanium at 5 V and 20 V created surfaces with titania nanotubes of 25 nm and 80 nm in size, respectively. To create hybrid surfaces with features on both the micron and nanoscale, acid etching of titanium was performed, which was then followed by anodisation at 5 V and 20 V, creating nanotubes on the micro acid etched surfaces, denoted as Micro/5VNT and Micro/20VNT. Controls of both polished (FlatTi) and acid etched Ti (AcidTi) were included in the study. Proliferation seemed to be largely unaffected by topography, although higher cell numbers were present on microsurfaces due to the increased surface area. The morphology of cells on the modified surfaces can be related to cellular differentiation [50], with MSCs located on modified surfaces being extended, polygonal, and osteoblast-like in appearance, noticeably different from cells found on FlatTi and AcidTi surfaces, which were small and resembled fibroblast-like cells. Regarding the ECM, after 2 weeks in culture, collagen formation was found to be denser on the modified surfaces in comparison to the controls. Mineralisation of the ECM was analysed by alizarin red staining, and it was found that all surfaces, apart from FlatTi, induced the formation of mineralised nodules. To support the ECM data, the expression of certain osteogenic genes was analysed. It was found that the topographies induced different levels of gene expression, although the 20VNT and Micro/20VNT produced the highest mRNA levels for important genes such as ALP, OCN, and BSP. This may be related to mechanotransduction signalling originating from the cellular extensions and modulated focal adhesions observed on the Micro/20VNT surface.

This observation of high levels of mRNA for important osteogenic genes on micro-/nanotitanium surfaces was also found by Mendonça et al. [62] using human MSCs. Using a technique of grit blasting and etching, Mendonça et al. created a topography on titanium discs which had both micro and nanofeatures (20–30 nm nanofeatures visible via SEM analysis). Titanium discs were grit blasted with 100 *μ*m aluminium oxide particles and then immersed in an H_2_SO_4_/H_2_O_2_ solution to give the surface its secondary nanofeatures, with the surface being referred to as Nano. A smooth Ti surface (S) and solely grit-blasted surface (Gb) were also studied. Real-time PCR was used to measure the level of mRNA for OCN, OPN, BSP, Runx2, ALP, and Osterix (OSX) at time points 3, 7, 14, and 28 days. It was found that at both 14 and 28 days, the Nanosurface was able to threefold upregulate important osteogenic genes Runx2 and OSX. The Nanosurface was also able to up regulate OCN (3-fold), ALP (38-fold), and BSP (76-fold), showing that the surface was capable of producing an enhanced osteogenic response in comparison to S and Gb surfaces. 

As in Zhao et al.'s [61] and Mendonça et al.'s [62] studies where the combination of both micron and nanosurface features (Micro/20VNTm and Nano) promoted an osteogenic response, in an attempt to better understand the role different sizes of surface features have on the osteogenic differentiation of MSCs, Khang et al. performed a study where sub-nano-, nano-, and submicron surfaces were analysed [63]. Pure titanium was deposited onto glass slips via an e-beam evaporator, and it was found that surfaces with features on the nano-submicron range proved to be the most influential on MSCs. Structures found on this 450 nm thick hybrid surface had heights of ~40 nm and widths of ~250 nm. Osteogenic genes Dlx5 and OSX were shown to be upregulated in mouse MSCs after 3 days in culture on the nano-submicron surface, alongside the upregulation of important osteogenic factors OC, BSP, OP, and collagen 1. Human MSC response to titanium surfaces was also studied, and the nano-submicron surface was again superior, with enhanced adhesion and proliferation at 6 and 72 hours. Levels of OC, OP, and osteonectin (ON) were also shown to be enhanced on the nano-submicron surface. Although the increased surface area and roughness of the nano-submicron surface can account for some of the enhanced osteogenic response, the increased surface structure width may also have caused elongation and extension of the MSCs, resulting in the transfer of mechanical stimuli in the cell cytoskeleton, to osteogenic biological response. 

Using a different surface modification technique known as surface mechanical attrition treatment (SMAT), Lai et al. investigated the behaviour of rat MSCs on treated titanium [64]. Through SMAT, titanium can be fabricated to have a nanostructured surface, created by serve plastic deformation of the top layer of a bulk material by means of repeated multidirectional impact of flying balls. Characterisation of the surfaces before and after SMAT treatment showed an increase in roughness from 7.29 nm *R*_*a*_ to 29.53 nm *R*_*a*_, accompanied by a decrease in water contact angle from 66.5° to 51.9°, which is a measure of surface energy, known to be important for bone integration [11, 12]. The cell morphology was visibly different on Ti- and SMAT-treated Ti (nano-Ti). Compared to MSCs on the native Ti surface, MSCs cultured on the nano-Ti were found to be well spread with well-developed actin stress fibers. The nano-Ti surface was also found to promote higher cell viability. To investigate the ability of the nano-Ti to stimulate osteogenic induction, Lai et al. looked at ALP activity, ECM mineralisation, osteogenic proteins, and mRNA expression of important osteogenic genes. It was found that the nano-Ti surface significantly enhanced all of these osteogenic factors, with the underlying mechanism thought to be the expression of OC, OPN, collagen 1, and Runx2. Lai et al. were able to provide an alternative method for creating nanotopographies on bulk titanium, with promising MSC adhesion, proliferation, and differentiation results. With further work, this surface modification technique may be capable of enhancing dental and orthopaedic implants.

## 6. Conclusion

Modified biomedical surfaces, which have been fabricated with topographical and chemical cues, have great potential to direct stem cell fate along a desired lineage. Specifically for dental and orthopaedic implants, an osteogenic response from MSCs is desired, stimulating rapid formation of new bone and osseointegration. In turn, this enhanced response will lead to shorter healing times of both dental and orthopaedic devices, due to enhanced MSC osteogenic differentiation leading to increased early bone apposition and integration into the implant. Similarly, rapid bone formation should increase early stage stability of the implant, preventing some failure mechanisms such as micromotion (a common issue following arthroplasty procedures such as THR). The use of topography offers the further advantage of removing the need for chemical stimuli to direct the desired differentiation which may carry numerous issues for *in vivo *application. Through modifying the surfaces of recognised biomedical implant materials, both the fields of dentistry and orthopaedics could have the potential to be revolutionised, by increasing implant efficacy via triggering the osteogenic differentiation of MSCs. 

This review has summarised the results of many studies showing that the osteogenic differentiation of MSCs can be achieved without the presence of chemical stimuli via numerous topographical surface modifications, including the use of nanopillars, nanotubes, nanopits, nanoislands, nanocraters, nanogrooves, and nanosawtooth structures, along with grit blasting/acid etching and SMAT techniques. What is also prevalent through analysing these studies is the wide variation of techniques used and occasionally dissimilar results. This field of research holds great potential for the possible reduction in healing times after implantation, although further study is required to clarify which techniques and specifications are optimal for triggering the osteogenic differentiation of MSCs. Further, to the *in vivo* possibilities of nano-patterned materials, there are also implications for the *ex vivo* expansion of stem cells for the use in reimplantation therapies. 

## References

[1] Kurtz SM, Ong KL, Schmier J (2007). Future clinical and economic impact of revision total hip and knee arthroplasty. *Journal of Bone and Joint Surgery. American*.

[2] Burke M, Revel PA (2008). Failure mechanisms in joint replacement. *Joint Replacement Technology*.

[3] Hench LL, Polak JM (2002). Third-generation biomedical materials. *Science*.

[4] Branemark PI (1983). Osseointegration and its experimental background. *The Journal of Prosthetic Dentistry*.

[5] Puleo DA, Nanci A (1999). Understanding and controlling the bone-implant interface. *Biomaterials*.

[6] Davies JE (1998). Mechanisms of endosseous integration. *International Journal of Prosthodontics*.

[7] Davies JE (2003). Understanding peri-implant endosseous healing. *Journal of Dental Education*.

[8] Tare RS, Babister JC, Kanczler J, Oreffo ROC (2008). Skeletal stem cells: phenotype, biology and environmental niches informing tissue regeneration. *Molecular and Cellular Endocrinology*.

[9] Geetha M, Singh AK, Asokamani R, Gogia AK (2009). Ti based biomaterials, the ultimate choice for orthopaedic implants—a review. *Progress in Materials Science*.

[10] Wennerberg A, Albrektsson T (2009). Effects of titanium surface topography on bone integration: a systematic review. *Clinical Oral Implants Research*.

[11] Zhao G, Schwartz Z, Wieland M (2005). High surface energy enhances cell response to titanium substrate microstructure. *Journal of Biomedical Materials Research Part A*.

[12] Lai HC, Zhuang LF, Liu X, Wieland M, Zhang ZY, Zhang ZY (2010). The influence of surface energy on early adherent events of osteoblast on titanium substrates. *Journal of Biomedical Materials Research Part A*.

[13] Buser D, Broggini N, Wieland M (2004). Enhanced bone apposition to a chemically modified SLA titanium surface. *Journal of Dental Research*.

[14] Schwarz F, Ferrari D, Herten M (2007). Effects of surface hydrophilicity and microtopography on early stages of soft and hard tissue integration at non-submerged titanium implants: an immunohistochemical study in dogs. *Journal of Periodontology*.

[15] Schwarz F, Herten M, Sager M, Wieland M, Dard M, Becker J (2007). Histological and immunohistochemical analysis of initial and early osseous integration at chemically modified and conventional SLA titanium implants: preliminary results of a pilot study in dogs. *Clinical Oral Implants Research*.

[16] Schwarz F, Herten M, Sager M, Wieland M, Dard M, Becker J (2007). Bone regeneration in dehiscence-type defects at chemically modified (SLActive) and conventional SLA titanium implants: a pilot study in dogs. *Journal of Clinical Periodontology*.

[17] Bornstein MM, Valderrama P, Jones AA, Wilson TG, Seibl R, Cochran DL (2008). Bone apposition around two different sandblasted and acid-etched titanium implant surfaces: a histomorphometric study in canine mandibles. *Clinical Oral Implants Research*.

[18] Lang NP, Salvi GE, Huynh-Ba G, Ivanovski S, Donos N, Bosshardt DD (2011). Early osseointegration to hydrophilic and hydrophobic implant surfaces in humans. *Clinical Oral Implants Research*.

[19] Donos N, Hamlet S, Lang NP (2011). Gene expression profile of osseointegration of a hydrophilic compared with a hydrophobic microrough implant surface. *Clinical Oral Implants Research*.

[20] Schwarz F, Wieland M, Schwartz Z (2009). Potential of chemically modified hydrophilic surface characteristics to support tissue integration of titanium dental implants. *Journal of Biomedical Materials Research Part B*.

[21] Wall I, Donos N, Carlqvist K, Jones F, Brett P (2009). Modified titanium surfaces promote accelerated osteogenic differentiation of mesenchymal stromal cells in vitro. *Bone*.

[22] Olivares-Navarrete R, Hyzy SL, Hutton DL (2010). Direct and indirect effects of microstructured titanium substrates on the induction of mesenchymal stem cell differentiation towards the osteoblast lineage. *Biomaterials*.

[23] Golub EE, Boesze-Battaglia K (2007). The role of alkaline phosphatase in mineralization. *Current Opinion in Orthopaedics*.

[24] Olivares-Navarrete R, Raz P, Zhao G (2008). Integrin *α*2*β*1 plays a critical role in osteoblast response to micron-scale surface structure and surface energy of titanium substrates. *Proceedings of the National Academy of Sciences of the United States of America*.

[25] Olivares-Navarrete R, Hyzy S, Wieland M, Boyan BD, Schwartz Z (2010). The roles of Wnt signaling modulators Dickkopf-1 (Dkk1) and Dickkopf-2 (Dkk2) and cell maturation state in osteogenesis on microstructured titanium surfaces. *Biomaterials*.

[26] Khan MR, Donos N, Salih V, Brett PM (2012). The enhanced modulation of key bone matrix components by modified titanium implant surfaces. *Bone*.

[27] Guilak F, Cohen DM, Estes BT, Gimble JM, Liedtke W, Chen CS (2009). Control of stem cell fate by physical interactions with the extracellular matrix. *Cell Stem Cell*.

[28] Engler AJ, Sen S, Sweeney HL, Discher DE (2006). Matrix elasticity directs stem cell lineage specification. *Cell*.

[29] Huebsch N, Arany PR, Mao AS (2010). Harnessing traction-mediated manipulation of the cell/matrix interface to control stem-cell fate. *Nature Materials*.

[30] Mamalis AA, Silvestros SS (2011). Analysis of osteoblastic gene expression in the early human mesenchymal cell response to a chemically modified implant surface: an in vitro study. *Clinical Oral Implants Research*.

[31] Bozec L, de Groot J, Odlyha M, Nicholls B, Horton MA (2005). Mineralised tissues as nanomaterials: analysis by atomic force microscopy. *IEE Proceedings Nanobiotechnology*.

[32] Bozec L, Horton MA (2006). Skeletal tissues as nanomaterials. *Journal of Materials Science: Materials in Medicine*.

[33] Dalby MJ, Gadegaard N, Tare R (2007). The control of human mesenchymal cell differentiation using nanoscale symmetry and disorder. *Nature Materials*.

[34] Dalby MJ, Andar A, Nag A (2008). Genomic expression of mesenchymal stem cells to altered nanoscale topographies. *Journal of the Royal Society Interface*.

[35] Dalby MJ, Pasqui D, Affrossman S (2004). Cell response to nano-islands produced by polymer demixing: a brief review. *IEE Proceedings Nanobiotechnology*.

[36] Biggs MJP, Richards RG, Gadegaard N (2009). Interactions with nanoscale topography: adhesion quantification and signal transduction in cells of osteogenic and multipotent lineage. *Journal of Biomedical Materials Research Part A*.

[37] Biggs MJP, Richards RG, Gadegaard N, Wilkinson CDW, Oreffo ROC, Dalby MJ (2009). The use of nanoscale topography to modulate the dynamics of adhesion formation in primary osteoblasts and ERK/MAPK signalling in STRO-1+ enriched skeletal stem cells. *Biomaterials*.

[38] Jaiswal RK, Jaiswal N, Bruder SP, Mbalaviele G, Marshak DR, Pittenger MF (2000). Adult human mesenchymal stem cell differentiation to the osteogenic or adipogenic lineage is regulated by mitogen-activated protein kinase. *Journal of Biological Chemistry*.

[39] Klees RF, Salasznyk RM, Kingsley K, Williams WA, Boskey A, Plopper GE (2005). Laminin-5 induces osteogenic gene expression in human mesenchymal stem cells through an ERK-dependent pathway. *Molecular Biology of the Cell*.

[40] Jadlowiec J, Koch H, Zhang X, Campbell PG, Seyedain M, Sfeir C (2004). Phosphophoryn regulates the gene expression and differentiation of NIH3T3, MC3T3-E1, and human mesenchymal stem cells via the integrin/MAPK signaling pathway. *Journal of Biological Chemistry*.

[41] Watari S, Hayashi K, Wood JA (2012). Modulation of osteogenic differentiation in hMSCs cells by submicron topographically-patterned ridges and grooves. *Biomaterials*.

[42] Augello A, de Bari C (2010). The regulation of differentiation in mesenchymal stem cells. *Human Gene Therapy*.

[43] Miyazono K, Miyazawa K (2002). Id: a target of BMP signaling. *Science's STKE*.

[44] Peng Y, Kang Q, Luo Q (2004). Inhibitor of DNA binding/differentiation helix-loop-helix proteins mediate bone morphogenetic protein-induced osteoblast differentiation of mesenchymal stem cells. *Journal of Biological Chemistry*.

[45] Mwenifumbo S, Stevens MM, Gonsalves KE, Halberstadt CR, Laurencin CT, Nair LS (2008). ECM interactions with cells from the macro- to nanoscale. *Biomedical Nanostructures*.

[46] Yim EKF, Pang SW, Leong KW (2007). Synthetic nanostructures inducing differentiation of human mesenchymal stem cells into neuronal lineage. *Experimental Cell Research*.

[47] Brammer KS, Choi C, Frandsen CJ, Oh S, Jin S (2011). Hydrophobic nanopillars initiate mesenchymal stem cell aggregation and osteo-differentiation. *Acta Biomaterialia*.

[48] Tang J, Peng R, Ding J (2010). The regulation of stem cell differentiation by cell-cell contact on micropatterned material surfaces. *Biomaterials*.

[49] Sjöström T, Dalby MJ, Hart A, Tare R, Oreffo ROC, Su B (2009). Fabrication of pillar-like titania nanostructures on titanium and their interactions with human skeletal stem cells. *Acta Biomaterialia*.

[50] Mathieu PS, Loboa EG (2012). Cytoskeletal and focal adhesion influences on mesenchymal stem cell shape, mechanical properties, and differentiation down osteogenic, adipogenic, and chondrogenic pathways. *Tissue Engineering Part B*.

[51] Biggs MJ, Dalby MJ (2010). Focal adhesions in osteoneogenesis. *Journal of Engineering in Medicine*.

[52] McNamara LE, Sjöström T, Burgess KE (2011). Skeletal stem cell physiology on functionally distinct titania nanotopographies. *Biomaterials*.

[53] Lavenus S, Trichet V, le Chevalier S, Hoornaert A, Louarn G, Layrolle P (2012). Cell differentiation and osseointegration influenced by nanoscale anodized titanium surfaces. *Nanomedicine*.

[54] Zhang W, Li Z, Liu Y (2012). Biofunctionalization of a titanium surface with a nano-sawtooth structure regulates the behavior of rat bone marrow mesenchymal stem cells. *International Journal of Nanomedicine*.

[55] Oh S, Brammer KS, Li YSJ (2009). Stem cell fate dictated solely by altered nanotube dimension. *Proceedings of the National Academy of Sciences of the United States of America*.

[56] Park J, Bauer S, von der Mark K, Schmuki P (2007). Nanosize and vitality: TiO_2_ nanotube diameter directs cell fate. *Nano Letters*.

[57] Park J, Bauer S, Schlegel KA, Neukam FW, von Mark KD, Schmuki P (2009). TiO_2_ nanotube surfaces: 15 nm—an optimal length scale of surface topography for cell adhesion and differentiation. *Small*.

[58] Park J, Bauer S, Pittrof A, Killian MS, Schmuki P, von der Mark K (2012). Synergistic control of mesenchymal stem cell differentiation by nanoscale surface geometry and immobilized growth factors on TiO_2_ nanotubes. *Small*.

[59] Lai M, Cai K, Zhao L, Chen X, Hou Y, Yang Z (2011). Surface functionalization of TiO_2_ nanotubes with bone morphogenetic protein 2 and its synergistic effect on the differentiation of mesenchymal stem cells. *Biomacromolecules*.

[60] Brammer KS, Frandsen CJ, Jin S (2012). TiO_2_ nanotubes for bone regeneration. *Trends in Biotechnology*.

[61] Zhao L, Liu L, Wu Z, Zhang Y, Chu PK (2012). Effects of micropitted/nanotubular titania topographies on bone mesenchymal stem cell osteogenic differentiation. *Biomaterials*.

[62] Mendonça G, Mendonça DBS, Aragão FJL, Cooper LF (2010). The combination of micron and nanotopography by H_2_SO_4_/H_2_O_2_ treatment and its effects on osteoblast-specific gene expression of hMSCs. *Journal of Biomedical Materials Research Part A*.

[63] Khang D, Choi J, Im YM (2012). Role of subnano-, nano- and submicron-surface features on osteoblast differentiation of bone marrow mesenchymal stem cells. *Biomaterials*.

[64] Lai M, Cai K, Hu Y, Yang X, Liu Q (2012). Regulation of the behaviors of mesenchymal stem cells by surface nanostructured titanium. *Colloids and Surfaces B*.

